# General recommendations paper on the management of older patients with cancer: the SEOM geriatric oncology task force’s position statement

**DOI:** 10.1007/s12094-018-1856-x

**Published:** 2018-04-09

**Authors:** R. Gironés Sarrió, M. Antonio Rebollo, M. J. Molina Garrido, C. Guillén-Ponce, R. Blanco, E. Gonzalez Flores, J. Saldaña, On behalf of the Spanish Working Group on Geriatric Oncology of the Spanish Society of Medical Oncology (SEOM)

**Affiliations:** 1 Medical Oncology Unit, Hospital Lluís Alcanyís, Crta Xàtiva A Silla Km 2, Xàtiva, 46800 Valencia, Spain; 2 Institut Català d’Oncologia, Barcelona, Spain; 3 Hospital General Virgen de la Luz, Cuenca, Spain; 4 Hospital Universitario Ramón and Cajal, Madrid, Spain; 5 Consorci Sanitari de Terrassa, Barcelona, Spain; 6 Hospital Virgen de las Nieves, Granada, Spain

**Keywords:** Geriatric oncology, Spanish society for medical oncology, Geriatric oncology task force, Aging

## Abstract

Population aging is associated with greater numbers of older people with cancer. Thanks to treatment advances, not only are more seniors diagnosed with cancer, but there are also more and more older cancer survivors. This upward trend will continue. Given the heterogeneity of aging, managing older patients with cancer poses a significant challenge for Medical Oncology. In Spain, a Geriatric Oncology Task Force has been set up within the framework of the Spanish Society for Medical Oncology (SEOM). With the aim of generating evidence and raising awareness, as well as helping medical oncologists in their training with respect to seniors with cancer, we have put together a series of basic management recommendations for this population. Many of the patients who are assessed in routine clinical practice in Oncology are older. CGA is the basic tool by means of which to evaluate older people with cancer and to understand their needs. Training and the correct use of recommendations regarding treatment for comorbidities and geriatric syndromes, support care, and drug–drug interactions and toxicities, including those of antineoplastic agents, as detailed in this article, will ensure that this population is properly managed.

## Introduction

The gradual aging of the world’s population, the greater risk of developing neoplasms at older ages, and the lack of scientific evidence have made the management of older individuals with cancer a tremendous care challenge [1]. Though significant strides have been made in recent years in awareness raising and research into aging and cancer, the care experience in Spain is limited to individual initiatives, with no national structure in place to approach this population cohort [2].

Aging is a highly disparate process, and as such, and given that chronological age does not always correspond with biological age, a patient’s date of birth should not be used as the sole discriminatory element when embarking on a diagnostic process or establishing the best treatment option for a specific neoplastic disease. One of the main difficulties lies in the very definition or diagnosis of aging. Despite the growing body of research in this field, thus far, we have no useful biological markers for care practices that enable us to determine a person’s biological age and aid us in treatment decision-making. At present, the only valid tool we have to ascertain the true biological status of an older individual with cancer is a comprehensive geriatric assessment (CGA) [3, 4]. While CGA is a widely used tool in various medical specialties, there is no consensus as to the scales to be used; furthermore, it calls for an investment of time and expertise that we are hard-pressed to assume within the reality of our care systems. There have been numerous proposals for simplified circuits or alternate variants in recent years, yet no consensus has been reached regarding a common strategy that would enable trials to be conducted to obtain scientific evidence and establish treatment protocols for each disease scenario [5].

In 2015, the Spanish Geriatric Oncology Group was created within the Spanish Society for Medical Oncology (SEOM) with the intention of analyzing the situation of geriatric oncology in Spain and to raise the awareness of professionals and political decision makers about the need to have a structured approach to this growing population. The main aim of the group is to approach geriatric oncology from an imminently pragmatic perspective; i.e., to provide the oncologist with a shared lexicon and simple, agreed upon tools that will be useful in real-world routine care and enable research to be conducted that focuses on responding to those situations in which we currently have no scientific evidence [2].

This document has been drafted with the purpose of providing general recommendations as to the evaluation and therapeutic management of the older patient with cancer. To this end, the first section has reviewed the concept of Geriatric Assessment, as well as the different scales that comprise it, focusing the second section on general recommendations to optimize management of seniors with cancer, regardless of the specific disease to be treated.

## Recommendations regarding evaluation and scales

Older individuals have less physiological reserve [6, 7] and, therefore, present a higher risk for iatrogenic complications [8]. They frequently suffer multiple diseases and geriatric syndromes, have a broad history of medications, and are often socially and functionally vulnerable. All this points to the fact that they require a comprehensive appraisal of their health status, including medical, cognitive, psychological, social, and functional aspects of daily life. Comprehensive Geriatric Assessment (CGA) systematically covers these key areas from a multidisciplinary perspective [9, 10] and to do so, must use valid, reliable, and sensitive measurement tools. It will thereby be capable of evaluating the changes individuals undergo over time against their baseline status. In this way, CGA will inform us as to the true situation of the person we are caring for, not only with respect to their age, but to their situation of biological vulnerability (whether they are robust or vigorous, pre-frail, frail, or dependent).

The scientific evidence available indicates that CGA in seniors with cancer provides more information than would otherwise be the case with only the physician’s intuition, making it an essential tool in Geriatric Oncology [11, 12]. Data from CGA lead to a modification of the treatment plan initially proposed by the specialist, and, therefore, supplies more information than the traditional assessment performed in younger patients [13–16]. The guidelines of the National Comprehensive Cancer Network (NCCN) recommend conducting CGA in individuals with cancer ≥ 65 years of age [17]. SIOG advises that all seniors seeking care undergo CGA [16], particularly in those with a treatment indication for their neoplasm.

The definition of who should undergo CGA will ultimately depend, too, on the availability of resources and on how care is organized at each center.

The transcendence of CGA in Geriatric Oncology links the advantages of CGA in older people with any disease with other specific advantages in seniors with cancer [18]: estimation of life expectancy [19–21], the risk of chemotherapy-derived toxicity [19, 22, 23] and early treatment suspension, or the possibility of functional decline [24, 25].

Insofar as the domains, a CGA should appraise is concerned, and SIOG drafted a consensus document in 2005, the latest update of which was published in 2014 [16]. In their consensus, they established that the domains that should appear in any CGA model are functional status, comorbidity, cognitive status, emotional state, social situation, nutritional status, and the presence of fatigue and geriatric syndromes [16]. Despite these recommendations, a recent publication-addressing seniors undergoing chemotherapy reflected that certain components of CGA are only ascertained in a small percentage of older patients [26].

As regards the scales to be used to appraise each domain, in SIOG’s latest consensus, no model was deemed superior to another [16]. Two consensus statements were drafted to determine the domains and scales to be used. One was carried out in United States [27] and the other was conducted at the international level [28]; in both cases, consensus was reached. In Spain, a consensus was also attained by SEOM’s task force on Geriatric Oncology. There is a Spanish consensus derived from the SEOM Geriatric Oncology Expert Committee with respect to the domains to be appraised by CGA and the scales to be used. The main conclusions regarding the domains and scales to be used in evaluating seniors with cancer are presented in Table 1 [2].

**Table 1:** Domains and scales recommended by the SEOM’s Geriatric Oncology Expert Committee

Domain	Scale
Functional	
ADL	Barthel scale
AIVD	Lawton–Brody Index
Functional status	Gait speed
Nutritional	MNA
Cognitive	Pfeiffer Questionnaire
Mood	Yesavage Questionnaire
Socio-familiar	Gijón social-familiar scale
Comorbidity	Charlson Index
Drug use	Number of medications
Geriatric syndromes	Insomnia
	Visual and auditory acuity
	Fecal and urinary incontinence
	Pressure sores
	Abuse

*MNA* mini-nutritional assessment, *ADL* activities of daily living, *IADL* instrumental activities of daily living

## General treatment management recommendations for seniors with cancer

Once the patient has been assessed as we have previous described and based on the circuits and resources available at each center, an individualized treatment proposal will be made.

Whatever the proposal is, there is a series of general considerations to be taken into account in treating any older patient with cancer. First of all, the individual’s comorbidities must be ascertained as well as how to intervene specifically with respect to the geriatric syndromes detected in the CGA. We must choose chemotherapy schedules or other cancer-specific treatments with the lowest toxicity and any treatment-induced effects must be prevented and aggressively treated as early as possible when, despite our best efforts, they do arise. Likewise, proper functional monitoring must be performed, depending on the person’s comorbidities and the guidelines used. Finally, adequate symptomatic control is imperative.

### Treatment and comorbidity stabilization

The presence of comorbidities can affect the treatment of cancer in seniors in very different ways [29]: comorbidities can influence how cancer behaves [30] and can hasten or delay its diagnosis; cancer treatment can worsen the comorbidity or entail unacceptable risk; the presence of comorbidities can condition life expectancy [31], and, finally, comorbidities can affect the results of oncological treatment [32].

### Specific intervention on the geriatric syndromes detected

As previously described, geriatric syndromes can be detected by CGA, evaluating the different biomedical and psychosocial domains as per protocol. Thus, we can often encounter a subacute functional decline that can sometimes improve through a multidisciplinary intervention. Discovering a situation of risk for falls will also enable us to implement preventive measures [33] and train the caregiver. The risk of suffering pressure sores due to immobilization is also not at all unusual and detecting it will also allow preventive measures to be put into place and an efficacious approach to be taken. The nutritional assessment incorporated into our appraisal can reveal patients who are at risk for malnutrition [34, 35], as well as those who can already be diagnosed as being malnourished. We will plan a proper nutritional intervention according to our care objectives (symptomatic improvement, cure, exclusively comfort care…) [36]. Cognitive impairments [37, 38] and confusional syndrome are also frequently diagnosed and we can generally provide efficacious treatment for them. Emotional disorders sleep cycle disturbances, and loss of sphincter control will be other geriatric syndromes we can act on. It is also very important to detect situations of caregiver burnout and offer support measures, which will surely impact the quality of life of both our seniors and their caregivers [39].

### Avoiding polypharmacy

Inappropriate drug prescription is especially common in older people and is associated with a higher risk of drug-related adverse events, more hospitalizations, and inappropriate resource use [40, 41]. We must also bear in mind the possible interactions between cancer drugs and any other medications the senior may be receiving [42]. We are all aware, for instance, of the interaction between different tyrosine-kinase inhibitors and proton pump inhibitors. Other usual situations are maintaining lipid-lowering treatments in patients with significant weight loss and short life expectancy, or continuing with antihypertensive medication in older individuals with multifactorial anemia that causes hypotension.

### Selection of low-toxicity treatment regimes

Systemic treatment of older individuals with cancer poses a challenge for the oncologist, given the variety of situations that must be attended to and the lack of published evidence in most cases. Only in recent years are studies being designed and specific results beginning to appear for this population. EORTC has prioritized this issue [43] and SIOG recommends that trials carried out in older patients evaluate their quality of life, functional status, and independence as priority objectives [44]. On the other hand, certain toxicities in particular, such as the neurotoxicity associated with some cytostatics, should be the object of study on their own [45, 46]. Furthermore, thromboembolic episodes appear to occur more frequently in older individuals who receive bevacizumab [47].

Finally, oral chemotherapy is an appealing option in seniors, due to better compliance in administering it and greater convenience compared to intravenous chemotherapy. Metronomic chemotherapy can represent a means of decreasing toxicity [48–50], thereby enhancing quality of life; moreover, several studies have pointed out the antiangiogenic and immunomodulating effects of this mode of administration [51].

Consequently, our general recommendations for this point would be:

- Insofar as possible, avoid cisplatin and paclitaxel combinations, given their neurotoxicity.
- Avoid anthracyclines in seniors with ejection fractions of less than 50% and consider alternatives, such as liposomal doxorubicin [52].
- Use drugs with a favorable toxicity profile: weekly vinorelbine, gemcitabine, or taxanes.
- Use capecitabine instead of 5-FU infusion.
- Exercise caution with the use of antiangiogenics.
- Avoid concurrent chemo-radiotherapy treatments.
- Consider the benefits of metronomic chemotherapy.

### Prevention and early treatment of the toxic effects of chemotherapy

Mucositis [53, 54]. In addition to impacting quality of life in people with cancer, oral mucositis influences treatment decisions and often necessitates dose reductions and delay or even treatment withdrawal. Being older and female are two risk factors for mucositis, for reasons as yet unknown. Deficient nutritional status, smoking, alcohol use, and periodontal disease are other patient-related risks.

Recommendations for mucositis prevention and treatment:

- Early hospitalization in individuals who develop dysphagia and/or diarrhea;
- Nutritional support;
- Oral prophylaxis and hygiene, and
- Attention to new drugs, such as palifermin (keratinocyte growth factors) [55, 56].

### Use of granulocytic colony-stimulating factors (G-CSF) and erythropoietin

Historically, when treatment intent was palliative, chemotherapy dose reduction was widespread to decrease the incidence of neutropenia in patients at risk. However, more recent publications maintain that G-CSF use would be justified if treatment intent is to prolong survival, even when it is not curative [57]. The National Cancer Comprehensive Network’s recommendations, as well as those of the 2015 American Society of Clinical Oncology (ASCO) [58], both advise primary prevention with G-CSF when the risk of febrile neutropenia surpasses 20%. However, these guidelines also recommend growth factors in people at “special” risk, including those over 65 years of age.

Indeed, already in 2001, SIOG recommended that colony-stimulating factors and erythropoietin be considered a fundamental element of treatment for senior cancer patients who are receiving chemotherapy, whether for radical or palliative purposes [59]. With respect to erythropoietin, it must be remembered that in older individuals, the symptoms that precede the anemia can quickly lead to a decline of their functional dependence.

In short, we believe that the use of colony-stimulating factors should be at least contemplated in all seniors who receive cytotoxic chemotherapy. We must also be especially alert to anemia secondary to chemotherapy and begin early treatment with erythropoietin as per guideline recommendations, particularly in patients with certain comorbidities (cardiac or respiratory), as anemia can have a major clinical and functional impact.

### Adequate symptom control

Together with the previously named support and recommendations, it is extremely important to ensure optimal symptomatic control by means of a multidisciplinary approach [60, 61]. The sphere of Palliative Care deals with more issues than simply controlling the individual’s symptoms. Treatment aims must be determined to enhance outcomes. Symptom management is similar in older and younger patients, but symptoms in seniors can be associated with complications that are both more common and more serious. In certain neoplasms, such as lung cancer, early palliative treatment associated with cancer-specific treatment has proven to go so far as to influence survival [62].

Our recommendation, therefore, is that any cancer individual that has no possibility for radical oncological treatment undergoes early evaluation by a Palliative Care team, especially if said individual is older, and for this assessment to be on-going throughout the entire process.

### Achieve adequate social support [63]

There is little agreement in the literature as to what constitutes adequate social support [64]. Some studies have attempted to quantify social support based on the number of relatives, for instance. Other works examine patients’ perception of the quality of their social support [65].

Despite all the difficulties incumbent in defining and quantifying social support, we believe that its necessity is evident and that it must be appraised and optimized for proper treatment planning for seniors with cancer.

## Conclusions

Many of the patients who are assessed in routine clinical practice in Oncology are older. CGA is the basic tool by means of which to evaluate older people with cancer and to understand their needs. Training and the correct use of recommendations regarding treatment for comorbidities and geriatric syndromes, support care, and drug–drug interactions and toxicities, including those of antineoplastic agents, as detailed in this article, will ensure that this population is properly managed.
